# Weighted Gene Co-expression Network Analysis of the Inflammatory Wnt Signaling Reveals Biomarkers Related to Bone Formation

**DOI:** 10.7759/cureus.63510

**Published:** 2024-06-30

**Authors:** Pradeep Kumar Yadalam, Ramya Ramadoss, Ramya Suresh

**Affiliations:** 1 Periodontics, Saveetha Dental College and Hospitals, Saveetha Institute of Medical and Technical Sciences, Saveetha University, Chennai, IND; 2 Oral Pathology and Oral Biology, Saveetha Dental College and Hospitals, Saveetha Institute of Medical and Technical Sciences, Saveetha University, Chennai, IND; 3 Oral Biology, Saveetha Dental College and Hospitals, Saveetha Institute of Medical and Technical Sciences, Saveetha University, Chennai, IND

**Keywords:** biomarkers, hub gene, bone formation, wnt signaling, wgcna

## Abstract

Background and aim

Osteocytes regulate bone metabolism and balance through various mechanisms, including the Wnt (Wingless-related integration site signal transduction) signaling pathway. Weighted gene co-expression network analysis (WGCNA) is a computational method to identify functionally related genes based on expression patterns, especially in the Wnt-beta-catenin and osteo-regenerative pathways. This study aims to analyze gene modules of the Wnt signaling pathway from WGCNA analysis.

Methods

The study used a microarray dataset from the GEO (GSE228306) to analyze differential gene expression in human primary monocytes. The study standardized datasets using Robust Multi-Array Average (RMA) expression measure and Integrated Differential Expression and Pathway (IDEP) analysis tool, building a co-expression network for group-specific component (GC) genes.

Results

The study uses WGCNA to identify co-expression modules with dysregulated mRNAs, revealing enrichment in Wnt-associated pathways and top hub-enriched genes like colony-stimulating factor 3 (CSF3), interleukin-6 (IL-6), IL-23 subunit alpha (IL23A), suppressor of cytokine signaling 1 (SOCS1), and C-C motif chemokine ligand 19 (CCL19).

Conclusion

WGCNA analysis of the Wnt signaling pathway will involve functional annotation, network visualization, validation, integration with other omics data, and addressing method limitations for better understanding.

## Introduction

Osteocytes maintain bone metabolism and homeostasis through several mechanisms [1]. Bone production depends on Wnt (Wingless-related integration site signal transduction), which affects mesenchymal stem cells and osteoblast differentiation [2]. The antibiotic doxycycline increases Wnt7b, a Wnt family protein important in osteoblast activity. Changes in Wnt1 signaling can influence osteoblast activity and bone health [3,4]. Oxidized phospholipids disrupt Wnt signaling, causing bone damage. MicroRNAs like miR-129-5p and miR-483-3p reduce osteoblast development, leading to osteoporosis. Dickkopf-1 (DKK-1) levels positively predict acute coronary syndrome (ACS) risk. Targeting tumor necrosis factor-related apoptosis-inducing ligand (TRAIL) and DKK-1 may be biomarkers for Alzheimer's disease and cognitive decline. Teriparatide raises serum DKK-1, suggesting a role in postmenopausal osteoporosis treatment [5,6].

Wnt signaling controls osteoblast and monocyte differentiation, and bone production [7]. It regulates monocyte fate and osteoblast differentiation [8]. Wnt signaling upregulates osteoblast development genes, and transcription factors like Runt-related transcription factor 2 (Runx2) and Osterix, directly and indirectly, regulate bone monocyte/macrophage lineage cells [9].

Weighted gene co-expression network analysis (WGCNA) is a novel method that constructs gene co-expression networks and identifies modules relevant to cancer biology [10]. It aims to find genes associated with colon cancer recurrence, study breast cancer progression, and develop prognostic predictors [11]. Computational weighted co-expression network analysis identifies functionally linked genes based on their expression patterns. This method could potentially discover genes co-expressed with Wnt-beta-catenin and osteo-regenerative pathway genes. By analyzing gene expression data across various tissues or cell types, researchers can identify co-expressed modules or groups of genes with similar expression patterns [12]. WGCNA analysis helps in identifying genes co-expressed with Wnt-beta-catenin and osteo-regenerative pathway genes, uncovering regulators, effectors, and modulators. It facilitates the exploration of complex biological networks and the analysis of Wnt signaling gene modules. Our aim is to analyze gene modules to identify functional hub genes using WGCNA.

## Materials and methods

One microarray dataset (GSE228306) from the National Center for Biotechnology Information (NCBI) Gene Expression Omnibus [13] was retrieved. This dataset features human primary monocytes expressing Wnt signaling components, which respond to Wnt-3a stimulation by increasing β-catenin protein levels. Interestingly, Wnt-3a treatment induces the secretion of cytokines and chemokines in circulating human monocytes, thereby enhancing monocyte migration.

Differential gene expression analysis

The study standardized datasets using the Robust Multi-Array Average (RMA) expression measure to ensure consistency and comparability among microarray investigations. This laid the foundation for later analyses utilizing the Integrated Differential Expression and Pathway (IDEP) analysis tool, version 2.0 [14].

The study used the RMA expression measure to standardize microarray datasets for differential gene expression analysis. The standardized datasets were then used for further analysis using the IDEP tool, a web-based gene expression data analysis platform. Statistical analysis was conducted using appropriate methods, comparing gene expression levels between experimental conditions or groups. Pathway analysis was also performed to identify differentially expressed genes and assess potential biological mechanisms. The IDEP tool provided various visualization options for interpretation, including plotting gene expression distributions and constructing pathway maps. This comprehensive analysis provided insights into differentially regulated genes and pathways.

WGCNA analysis

Using WGCNA, the study generated a co-expression network for group-specific component (GC) genes by converting gene expression matrices into adjacency matrices, topological overlap and dissimilarity topological overlap matrices (TOMs), and mRNA similarity matrices. The process involved obtaining gene expression data, transforming it into adjacency matrices, transforming it into a TOM, generating a dissimilarity TOM, and generating mRNA similarity matrices. These matrices provided a comprehensive representation of co-expression patterns and similarities between GC genes, allowing for further analysis and interpretation of the network. The study's findings provide valuable insights into the role of genes in the Wnt pathway [15,16].

Gene ontology

Biological processes, molecular roles, cellular components, and Kyoto Encyclopedia of Genes and Genomes (KEGG) pathways for mRNAs were identified using the IDEP program, which was used for gene ontology and KEGG pathway enrichment analysis of hub clusters [17].

The study used the IDEP program to identify biological processes, molecular roles, cellular components, and KEGG pathways in the co-expression network of the Wnt pathway [18]. This process involved identifying hub clusters, which are highly interconnected groups of genes. The IDEP program utilized the input data for enrichment analysis of the uploaded gene set. It also performed gene ontology enrichment analysis to identify enriched biological processes, molecular roles, and cellular components, as well as KEGG pathway enrichment analysis to identify enriched pathways. Statistical analysis using Fisher's exact or hypergeometric test was conducted to determine the significance of the enrichment results. The study provided insights into the functional characteristics of genes and their potential roles within the hub clusters.

## Results

**Figure 1:**
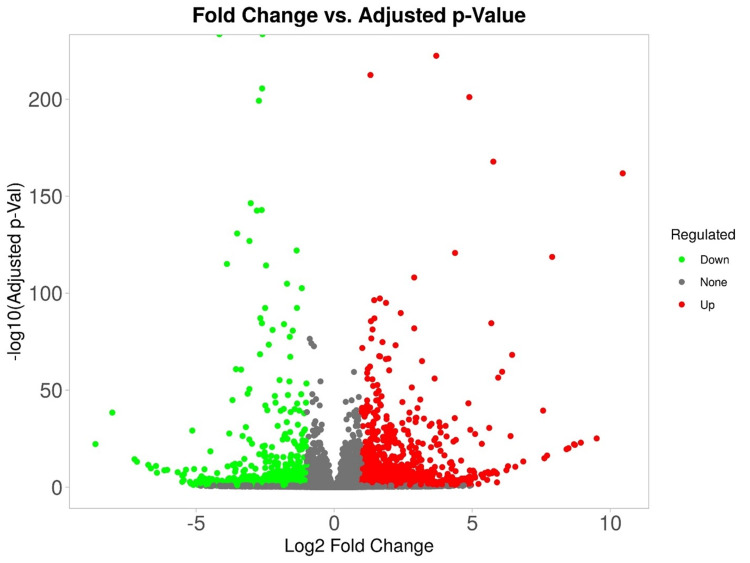
A volcano of upregulated genes as red and downregulated genes as green, with the most significant DEGs DEGs: Differentially expressed genes

The study analyzed a specific group of genes in modules, revealing a significant cluster of pathways. The pathway analysis revealed a high false discovery rate (FDR) (1.13E-05) for interleukin-10 (IL-10) production, indicating significant enrichment of genes involved in this process. The pathway also showed positive regulation of T-cell proliferation, response to the bacterium, and positive regulation of IL-10 production. Additionally, the pathway exhibited responses to external stimuli, mononuclear cell proliferation, and positive regulation of lymphocyte proliferation. The fold enrichment values indicated that the genes in each pathway were overrepresented compared to what would be expected by chance (Figure 1). 

**Figure 2:**
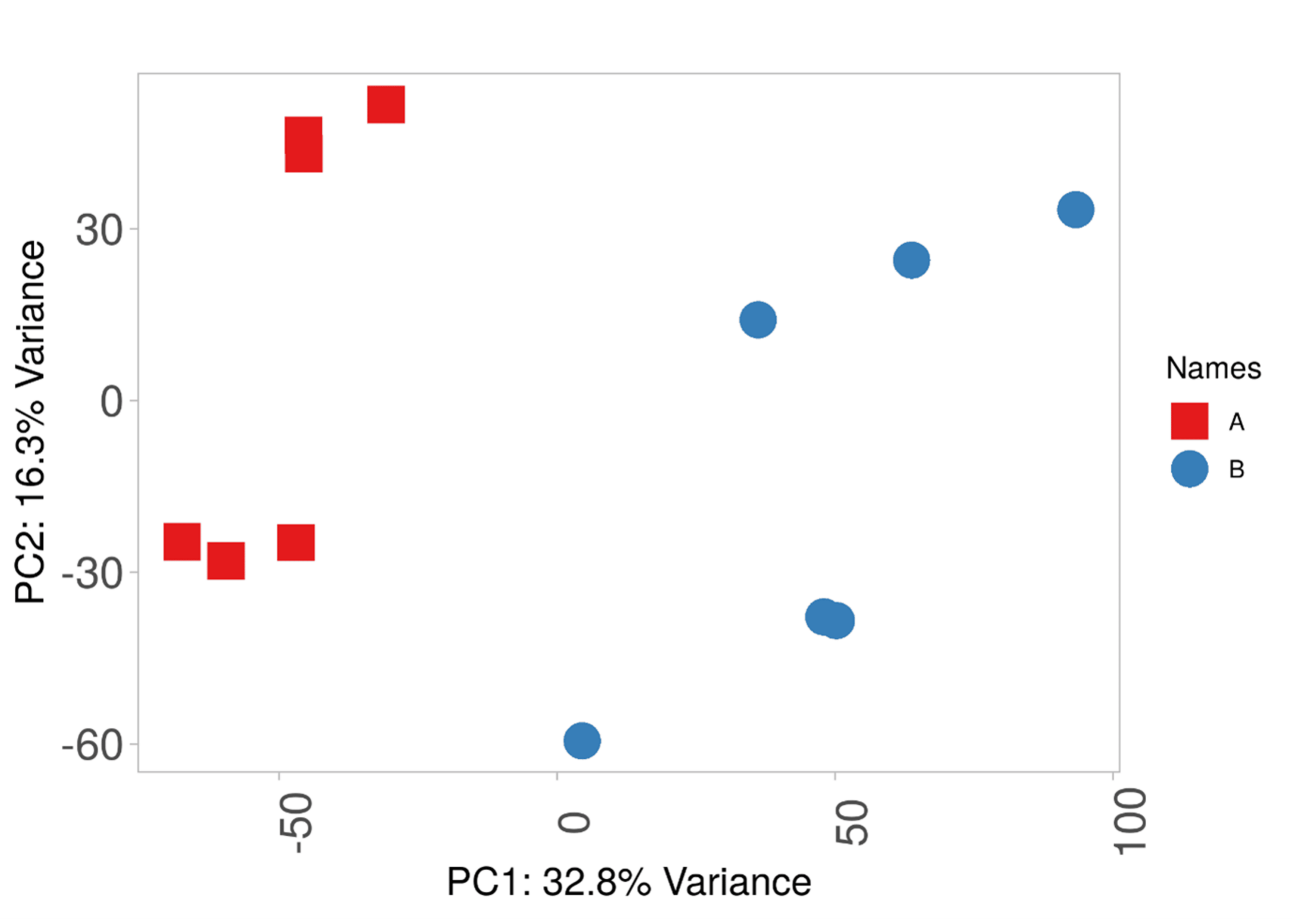
Principal component analysis with DEGs DEGs: Differentially expressed genes; PC: Principal component

Principal component (PC) analyses were performed with differentially expressed genes; in particular, PC1 explains 32.8% of the variance in gene expression (Figure 2).

**Figure 3:**
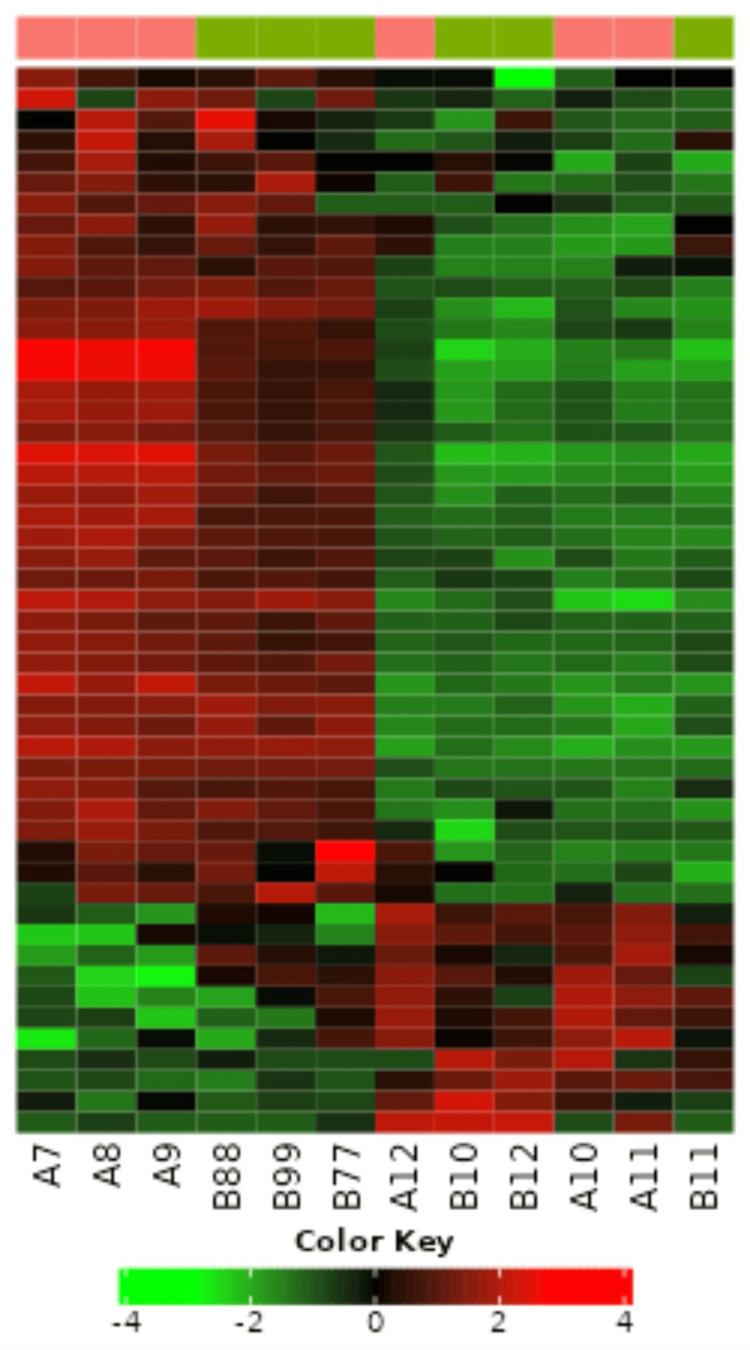
Heat map graphic

The topological overlap matrix of all the genes analyzed is shown in Figure 3. 

**Figure 4:**
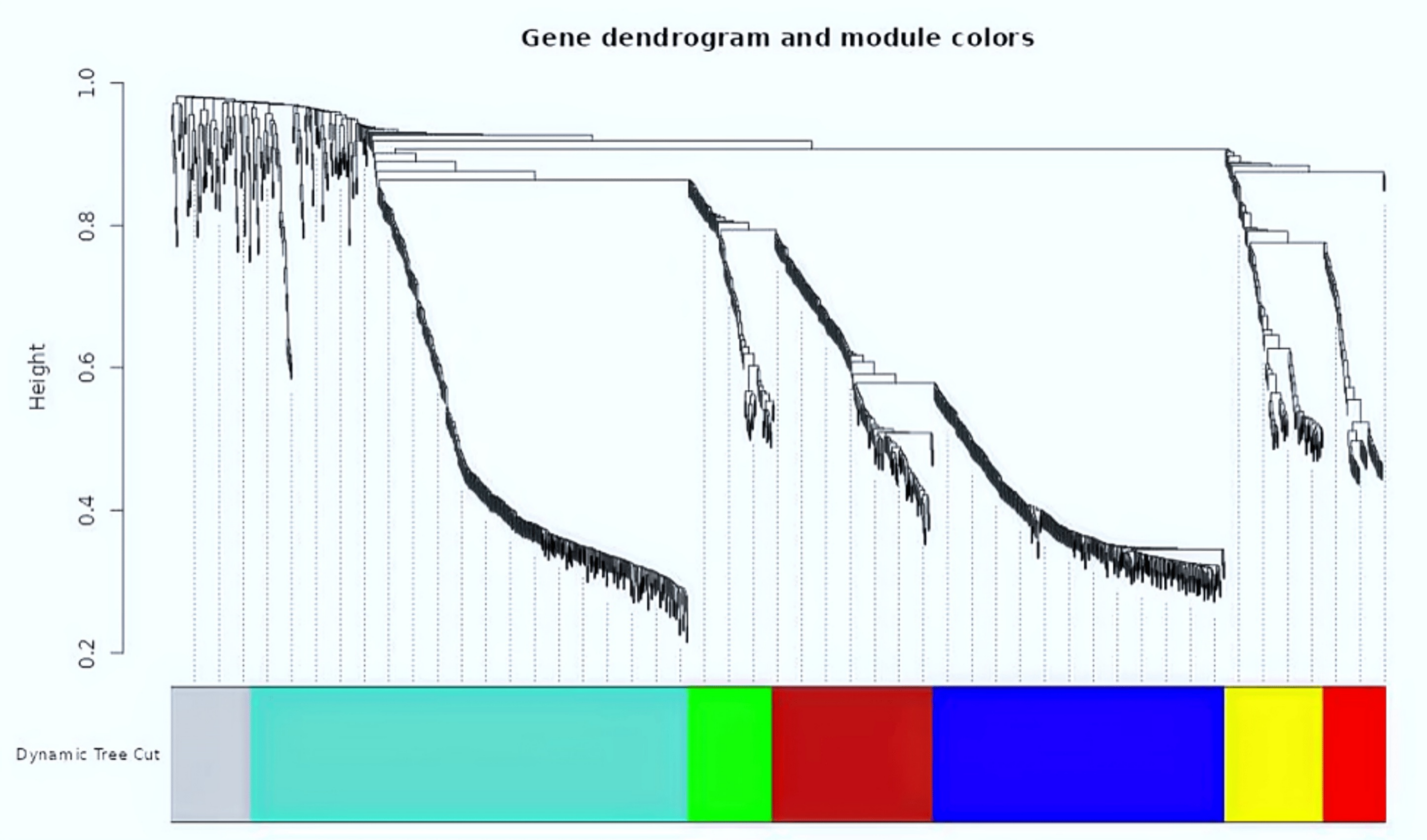
Dendrogram, created through dissimilarity clustering, displays various modules in different colors, each containing tightly interconnected genes, with colored lines indicating their specific modules

Figure 4 shows a clustering dendrogram of genes. The color bands provide a simple visual comparison of the module (and merged dynamically) assignments, based on the dynamic tree-cutting method.

**Figure 5:**
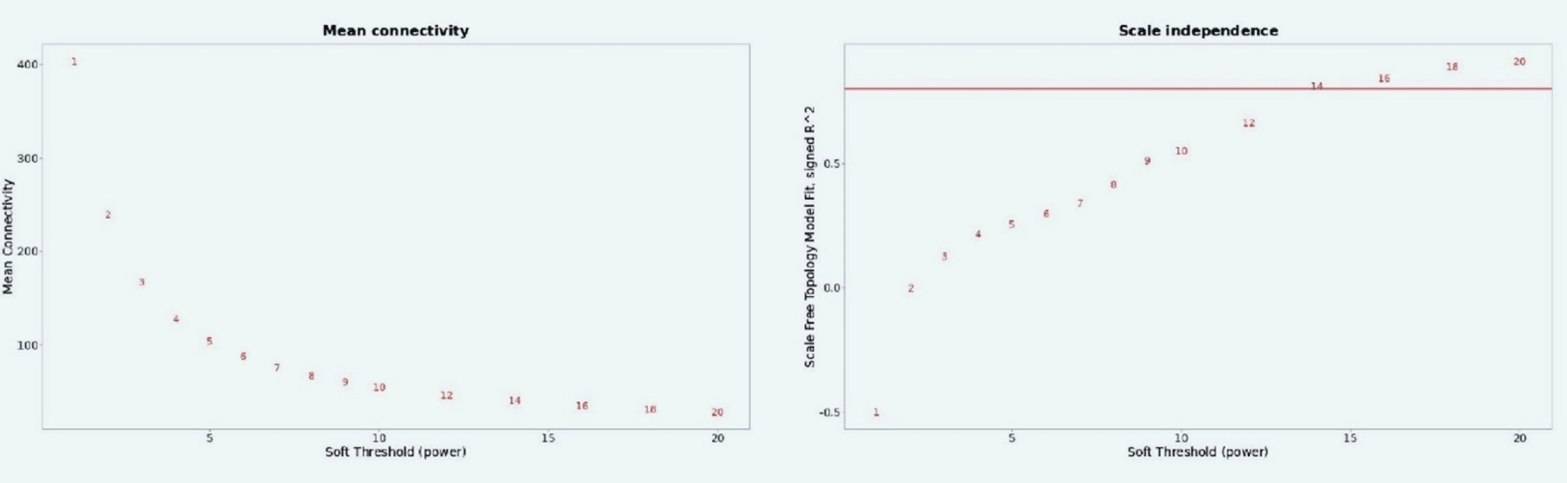
An analysis that reveals a scale-free topology fitting index R2: Correlation coefficient

Scale-free topology fitting index (R2) and mean connectivity for various soft threshold powers, with an R2 value of 14, are shown in Figure 5.

**Figure 6:**
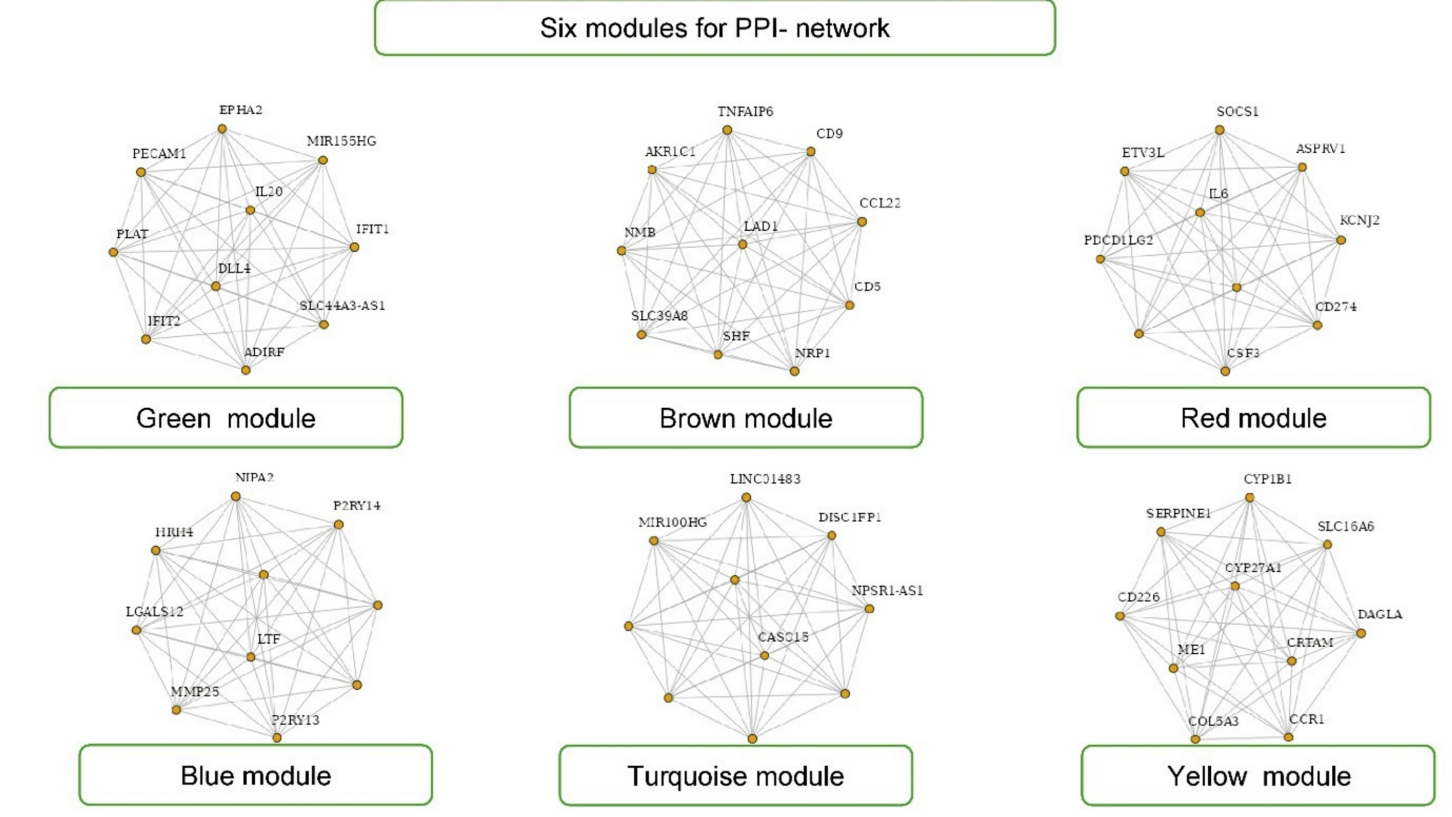
Top six modules with hub genes PPI: Protein-protein interaction

The top six modules for the protein-protein interaction (PPI) network with 10 hub genes from the WGCNA analysis are shown in Figure 6.

The table analyzes differentially expressed genes in a specific cluster, revealing significant FDRs and fold enrichment in pathways like Janus kinase-signal transducer (JAK-STAT), cytokine-cytokine receptor interaction, malaria, inflammation bowel disease, viral protein interaction, hematopoietic cell lineage, pertussis, COVID-19, rheumatoid arthritis, and IL-17 signaling pathway aiding researchers in understanding their potential biological processes (Tables 1-2). 

**Table 1:** Enriched cluster of the genes FDR: False discovery rate; JAK-STAT: Janus kinase-signal transducer

SI No.	Group	FDR	Fold enriched	Pathway
1	Cluster	3.27E-03	18.17090909	JAK-STAT signaling pathway
2	Cluster	8.39E-03	11.95454545	Cytokine-cytokine receptor interaction
3	Cluster	4.21E-02	31.32915361	Malaria
4	Cluster	5.99E-02	22.71363636	Inflammatory bowel disease
5	Cluster	6.29E-02	14.89418778	Viral protein interaction with cytokine and cytokine receptor
6	Cluster	6.29E-02	14.89418778	Hematopoietic cell lineage
7	Cluster	6.29E-02	16.82491582	Pertussis
8	Cluster	6.29E-02	8.209748083	Coronavirus disease - COVID-19
9	Cluster	6.29E-02	14.65395894	Rheumatoid arthritis
10	Cluster	6.39E-02	13.76584022	IL-17 signaling pathway

**Table 2:** Enriched genes of the hub modules

SI No.	Group	FDR	Fold enriched	Pathway
1	Cluster	1.13E-05	59.05	Interleukin-10 production
2	Cluster	1.13E-05	59.05	Regulation of interleukin-10 production
3	Cluster	8.34E-05	36.90625	Positive regulation of T cell proliferation
4	Cluster	9.04E-05	10.92956298	Response to bacterium
5	Cluster	1.02E-04	64.41818182	Positive regulation of interleukin-10 production
6	Cluster	1.21E-04	29.85674157	CD4-positive alpha-beta T cell activation
7	Cluster	1.48E-04	17.52032967	Positive regulation of T cell activation
8	Cluster	1.58E-04	4.129617454	Response to external stimulus
9	Cluster	1.58E-04	25.06839623	Positive regulation of mononuclear cell proliferation
10	Cluster	1.58E-04	25.79854369	Positive regulation of lymphocyte proliferation

## Discussion

Bone formation and stem cell maintenance depend on the Wnt-β-catenin pathway [19,20]. Wnt proteins block β-catenin breakdown and promote its cytoplasmic accumulation, enabling interaction with transcription factors and gene activation [21,22]. Human diseases, including cancer and developmental disorders such as familial exudative vitreoretinopathy and osteoporosis-pseudoglioma syndrome, have been linked to dysregulation of the Wnt-β-catenin pathway [23]. In regenerative medicine, manipulating the Wnt-β-catenin pathway can impact stem cell behavior and tissue regeneration. Many medications and investigational chemicals have modulated the pathway's activity to suppress cancer cells or activate tissue repair and regeneration [24].

Bioinformatics tool WGCNA identifies gene modules and their connections with characteristics or diseases. It can be used to study gene co-expression patterns in the Wnt signaling pathway and their possible effects on biological processes and illnesses. Researchers can build a co-expression network using a huge dataset to find highly co-expressed Wnt pathway gene modules. This information may identify pathway regulatory mechanisms and functional linkages [25]. WGCNA can reveal disease mechanisms, potential drug targets, and therapeutic development when combined with other datasets or functional annotation databases. This analysis found colony-stimulating factor 3 (CSF3), IL-6, IL-23 subunit alpha (IL-23A), suppressor of cytokine signaling 1 (SOCS1), and C-C motif chemokine ligand 19 (CCL19) to be hub-enriched genes [26].

WGCNA-enriched genes include one study that found the CSF3-low-expression group is strong in Wnt signaling. G-CSF phosphorylates Akt receptors to reduce glucose-regulated protein 78 (GRP 78) and endoplasmic reticulum (ER) stress apoptotic pathway proteins, and regulate neutrophil and granulocyte production [27]. When coupled with TNFα and DKK-1, IL-6 suppresses Wnt signaling and responsiveness in human synoviocytes, reducing IL-6 mRNA boosts osteogenesis, mitigates TNFα's detrimental effects, and improves bone matrix mineralization. Bone creation, development, and remodeling require Wnt signaling; it speeds up bone healing [28]. Inhibiting Wnt signaling improves fracture site bone growth, but targeting IL-23 signaling may damage joints and bone. Mutations in β-catenin can hinder healing; bone production requires Wnt signaling. Therefore, blocking it improves fracture repair. STAT1/3 inhibitor and SOCS1 may also enhance regeneration [29].

WGCNA co-expression networks can be used to study the Wnt signaling pathway. Functional annotation, network visualization, validation, and omics data integration are possible. Functional annotation helps comprehend gene-related biological processes and pathways in each module. Network visualization tools can discover hub genes and central nodes [30]. Validation and experimental validation can verify gene module importance and reliability. WGCNA assumes that co-expressed genes are functionally linked, relying on the quality and availability of gene expression datasets, but it has limitations. To better understand the Wnt pathway's involvement in biology, WGCNA analysis will follow these steps. Furthermore, sample size, biological and technical variability, heterogeneity, platform biases, missing data, difficulties integrating data, and a lack of functional annotations are some of the constraints that gene expression databases must contend with. These elements have the potential to lower statistical power, add noise, obstruct interpretation, and affect study comparability. Analysis can also be hindered by missing data brought about by technical problems or insufficient sample size.

## Conclusions

WGCNA analysis is a method that employs functional annotation, network visualization, validation, and integration with other omics data to study the Wnt signaling pathway. This approach helps identify key biological processes, molecular roles, and cellular components associated with the Wnt pathway for bone formation. Network visualization aids in identifying crucial hub genes or clusters, while experimental validation ensures the accuracy and reliability of the findings. Integrating WGCNA results with other omics data, such as proteomics or epigenomics, further enhances the understanding of the pathway's regulation and impact on cellular processes. However, the approach has limitations, such as assuming that genes with similar expression patterns are functionally related. Additional experimental evidence and functional annotations are necessary to validate these relationships. The WGCNA analysis approach offers a valuable framework for studying the Wnt signaling pathway, but researchers should consider these limitations and support findings with complementary experimental evidence.

## References

[1] Majidinia M, Sadeghpour A, Yousefi B (2018). The roles of signaling pathways in bone repair and regeneration. J Cell Physiol.

[2] Cui J, Shibata Y, Zhu T, Zhou J, Zhang J (2022). Osteocytes in bone aging: advances, challenges, and future perspectives. Ageing Res Rev.

[3] Baron R, Kneissel M (2013). WNT signaling in bone homeostasis and disease: from human mutations to treatments. Nat Med.

[4] Shen G, Ren H, Shang Q (2020). Foxf1 knockdown promotes BMSC osteogenesis in part by activating the Wnt/β-catenin signalling pathway and prevents ovariectomy-induced bone loss. EBioMedicine.

[5] Lojk J, Marc J (2021). Roles of non-canonical Wnt signalling pathways in bone biology. Int J Mol Sci.

[6] Kovács B, Vajda E, Nagy EE (2019). Regulatory effects and interactions of the Wnt and OPG-RANKL-RANK signaling at the bone-cartilage interface in osteoarthritis. Int J Mol Sci.

[7] MacDonald BT, He X (2012). Frizzled and LRP5/6 receptors for Wnt/β-catenin signaling. Cold Spring Harb Perspect Biol.

[8] Wo D, Peng J, Ren DN (2016). Opposing roles of Wnt inhibitors IGFBP-4 and DKK1 in cardiac ischemia by differential targeting of LRP5/6 and β-catenin. Circulation.

[9] Ahn VE, Chu ML, Choi HJ, Tran D, Abo A, Weis WI (2011). Structural basis of Wnt signaling inhibition by Dickkopf binding to LRP5/6. Dev Cell.

[10] Marini F, Giusti F, Palmini G, Brandi ML (2023). Role of Wnt signaling and sclerostin in bone and as therapeutic targets in skeletal disorders. Osteoporos Int.

[11] Quan Q, Xiong X, Wu S, Yu M (2021). Identification of immune-related key genes in ovarian cancer based on WGCNA. Front Genet.

[12] Zeng J, Lai C, Luo J, Li L (2023). Functional investigation and two-sample Mendelian randomization study of neuropathic pain hub genes obtained by WGCNA analysis. Front Neurosci.

[13] Tian Z, He W, Tang J, Liao X, Yang Q, Wu Y, Wu G (2020). Identification of important modules and biomarkers in breast cancer based on WGCNA. Onco Targets Ther.

[14] Liu K, Chen S, Lu R (2021). Identification of important genes related to ferroptosis and hypoxia in acute myocardial infarction based on WGCNA. Bioengineered.

[15] Lin W, Wang Y, Chen Y, Wang Q, Gu Z, Zhu Y (2021). Role of calcium signaling pathway-related gene regulatory networks in ischemic stroke based on multiple WGCNA and single-cell analysis. Oxid Med Cell Longev.

[16] Barrett T, Wilhite SE, Ledoux P (2013). NCBI GEO: archive for functional genomics data sets--update. Nucleic Acids Res.

[17] Edgar R, Domrachev M, Lash AE (2002). Gene expression omnibus: NCBI gene expression and hybridization array data repository. Nucleic Acids Res.

[18] Ge SX, Son EW, Yao R (2018). iDEP: an integrated web application for differential expression and pathway analysis of RNA-Seq data. BMC Bioinformatics.

[19] Galli C, Passeri G, Macaluso GM (2010). Osteocytes and WNT: the mechanical control of bone formation. J Dent Res.

[20] Chen G, Deng C, Li YP (2012). TGF-β and BMP signaling in osteoblast differentiation and bone formation. Int J Biol Sci.

[21] Tan Z, Ding N, Lu H, Kessler JA, Kan L (2019). Wnt signaling in physiological and pathological bone formation. Histol Histopathol.

[22] Kulyk WM, Reichert C (1992). Staurosporine, a protein kinase inhibitor, stimulates cartilage differentiation by embryonic facial mesenchyme. J Craniofac Genet Dev Biol.

[23] Dong B, Hiasa M, Higa Y (2022). Osteoblast/osteocyte-derived interleukin-11 regulates osteogenesis and systemic adipogenesis. Nat Commun.

[24] Hexner EO, Serdikoff C, Jan M (2008). Lestaurtinib (CEP701) is a JAK2 inhibitor that suppresses JAK2/STAT5 signaling and the proliferation of primary erythroid cells from patients with myeloproliferative disorders. Blood.

[25] Clevers H, Nusse R (2012). Wnt/β-catenin signaling and disease. Cell.

[26] Zhang X, Dong N, Hu X (2023). Wnt/β-catenin signaling inhibitors. Curr Top Med Chem.

[27] Zhang Y, Jing Z, Cao X (2022). SOCS1, the feedback regulator of STAT1/3, inhibits the osteogenic differentiation of rat bone marrow mesenchymal stem cells. Gene.

[28] Malysheva K, de Rooij K, Lowik CW, Baeten DL, Rose-John S, Stoika R, Korchynskyi O (2016). Interleukin 6/Wnt interactions in rheumatoid arthritis: interleukin 6 inhibits Wnt signaling in synovial fibroblasts and osteoblasts. Croat Med J.

[29] Razawy W, van Driel M, Lubberts E (2018). The role of IL-23 receptor signaling in inflammation-mediated erosive autoimmune arthritis and bone remodeling. Eur J Immunol.

[30] Gao Y, Chen N, Fu Z, Zhang Q (13). Progress of Wnt signaling pathway in osteoporosis. Biomolecules.

